# First-Principles Study of 3*R*-MoS_2_ for High-Capacity and Stable Aluminum Ion Batteries Cathode Material

**DOI:** 10.3390/molecules29225433

**Published:** 2024-11-18

**Authors:** Bin Wang, Tao Deng, Quan Zhou, Chaoyang Zhang, Xingbao Lu, Renqian Tao

**Affiliations:** 1 School of Physics and Electronic Engineering, Xinxiang University, Xinxiang 453003, China; dengtaoxuyiyin@163.com (T.D.);; 2 School of Mechanical Engineering, Chengdu University, Chengdu 610106, China; 3 Henan Province Engineering Research Center of New Energy Storage System, Xinxiang University, Xinxiang 453003, China; 4 School of Physical Science and Technology, Lanzhou University, Lanzhou 730000, China; taorg@lzu.edu.cn

**Keywords:** 3*R*-MoS_2_, cathode, aluminum ion battery, first-principles, intercalation mechanism

## Abstract

Currently, exploring high-capacity, stable cathode materials remains a major challenge for rechargeable Aluminum-ion batteries (AIBs). As an intercalator for rechargeable AIBs, Al^3+^ produces three times the capacity of AlCl_4_^−^ when the same number of anions is inserted. However, the cathode material capable of producing Al^3+^ intercalation is not a graphite material with AlCl_4_^−^ intercalation but a transition metal sulfide material with polar bonding. In this paper, the insertion mechanism of Al^3+^ in 3*R*-MoS_2_ is investigated using first-principles calculations. It is found that Al^3+^ tends to insert into different interlayer positions at the same time rather than occupying one layer before inserting into another, which is different from the insertion mechanism of AlCl_4_^−^ in graphite. Ab initio, molecular dynamics calculations revealed that Al^3+^ was able to stabilize the insertion of 3*R*-MoS_2_. Diffusion barriers indicate that Al^3+^ preferentially migrates to nearby stabilization sites in diffusion pathway studies. According to the calculation, the theoretical maximum specific capacity of Al^3+^ intercalated 3*R*-MoS_2_ reached 502.30 mAg h^−1^, and the average voltage of the intercalation was in the range of 0.75–0.96 V. Therefore, 3*R*-MoS_2_ is a very promising cathode material for AIBs.

## 1. Introduction

Energy storage batteries have always been the focus of attention. Currently, metal-ion batteries have attracted significant attention from researchers due to their small size and high efficiency. Lithium-ion batteries (LIBs) are a widely utilized energy storage solution due to their high energy density, charge/discharge voltage, and capacity [1,2]. However, in comparison to other metal resources, lithium metal reserves are relatively limited (0.0017 wt%), and the cost of LIBs has been on an upward trajectory. Furthermore, safety concerns, such as the formation of lithium dendrites, are becoming increasingly prominent [3,4,5,6,7]. In this context, Aluminum-ion batteries (AIBs) have emerged as a highly promising option due to their abundance of energy storage, high energy density, and extensive reserves [8]. However, AIBs still lack cathode materials with high capacity and stability [9,10].

In 2015, Dai et al. developed ultra-fast charging AIBs comprising an anode of metallic aluminum and a cathode of three-dimensional foam graphite, a material that exhibits an ultra-fast charging rate but a low capacity [11]. This was due to the intercalation and deintercalation of AlCl_4_^−^ in the electrode material, which did not utilize the properties of Al^3+^. Furthermore, the large size of the chloroaluminum anion causes volume expansion [12]. Al^3+^ carries more charge than AlCl_4_^−^, which means that when the same amount of AlCl_4_^−^ is inserted, Al^3+^ can produce more capacity. Currently, transition metal oxides with polar bonds are considered potential cathode materials for Al^3+^ inserted [13]. In 2011, Jayaprakash et al. reported for the first time that V_2_O_5_ nanowires could serve as cathode materials for Al^3+^ intercalation. The cell obtained a capacity retention of 273 mAh g^−1^ after 20 cycles at a current density of 125 mA g^−1^. However, the high charge density on the Al^3+^ surface produces strong Coulombic ion−lattice interactions that hinder the diffusion of Al^3+^ in the lattice, limiting the rate performance and cycling stability of the AIBs [13,14]. To reduce this strong Coulombic ion–lattice interaction, transition metal sulfides (TMDs) with weak Coulombic interactions have been used for extensive studies [15,16]. In 2018, Li et al. first prepared a rechargeable aluminum ion battery consisting of MoS_2_ microsphere cathode, aluminum anode, and ionic liquid electrolyte, demonstrating the feasibility of MoS_2_ as a cathode for AIBs [17]. In 2019, Tu et al. synthesized hierarchical flower-like MoS_2_ microspheres as AIBs cathodes by a simple hydrothermal method and showed that the structure of MoS_2_ was stabilized during Al^3+^ intercalation/deintercalation by density functional theory calculations [18]. All the above studies are based on 2*H*-MoS_2_. However, 3*R*-MoS_2_, which is also a semiconductor phase with a stable thermodynamic ABC stacking mode (unlike 2*H*-MoS_2_ AB stacking), lacks studies specifically on electrochemical behavior [19,20].

This study systematically investigates the structural stability, electronic properties, theoretical capacity, and average voltage of Al^3+^ intercalation/deintercalation 3*R*-MoS_2_ electrodes using first-principles calculations. Furthermore, the thermal stability of 3*R*-MoS_2_ is examined using the Ab Initio Molecular Dynamics (AIMD) method, and the diffusion path of Al^3+^ in the 3*R*-MoS_2_ layer is investigated in detail. Our findings were benchmarked against the most recent experimental data on AIBs. Furthermore, we conducted a detailed investigation of the Al^3+^ inserted MoS_2_ system to elucidate the cation insertion mechanism in MoS_2_, with the objective of facilitating the design of superior electrodes in the future.

## 2. Results and Discussions

### 2.1. Single Al^3+^ Inserted in 3R-MoS_2_

**Figure 1:**
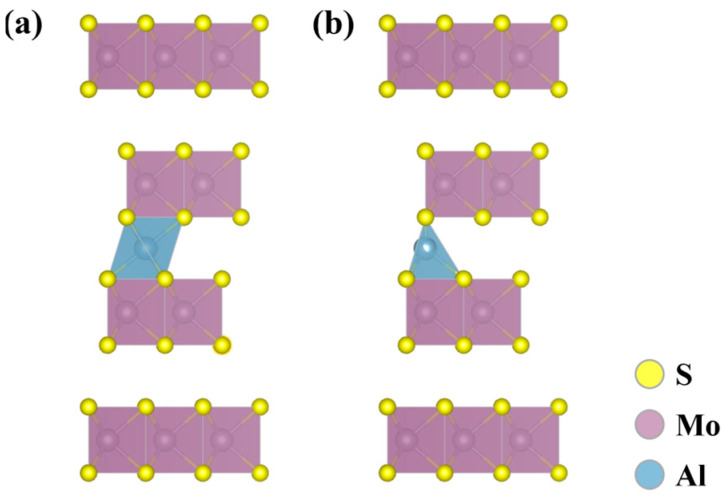
Two preferred inserted positions of Al^3+^ in 3*R*-MoS_2_ (**a**) at position A and (**b**) at position B.

A comprehensive examination of all potential inserted locations for Al^3+^ within MoS_2_ has been conducted. Two preferred insertion positions of Al^3+^ in 3*R*-MoS_2_ were envisioned based on the study of potassium ion intercalation at the 2*H*-MoS_2_ site (Figure 1): positions A and B (named PA and PB) [21]. At PA, the Al^3+^ is situated within an octahedron comprising six S atoms. It occupies a bridging position between two nonbonding S atoms, thereby forming six Al–S bonds. At PB, the Al^3+^ is situated within a tetrahedron of four S atoms, and Al^3+^ also occupies a bridging position between two non−bonded S atoms, forming four Al–S bonds. According to binding energy studies, these two positions are very favorable, and the two energies are very close. (Table S1). The binding energy for PA is less than that of PB, indicating that PA is the more stable site for Al^3+^ intercalation (consistent with the stabilization site in 2*H*-MoS_2_ [18]). The subsequent section of this study focuses on the insertion of an Al^3+^ at PA. The energies associated with the intercalation of an Al^3+^ into the top, middle, and bottom layers of PA exhibit minimal variation. This indicates that the initial insertion of the Al^3+^ is random with respect to the layer in question.

The thermal stability of Al^3+^ inserted into the 3*R*-MoS_2_ structure was investigated with AIMD (Figure S1). The most stable PA was selected for the simulation. Initially, the structure was subjected to a heating process, reaching a temperature of 300 K over a time interval of 1 fs to 5 ps. It was observed that the ABC stacking of 3*R*-MoS_2_ remained unaltered from the snapshot taken at 5 ps. The structure remained intact, and the embedded Al^3+^ exhibited minimal displacement within the PA. Furthermore, simulations were conducted using the NVT system at temperatures of 400 K, 500 K, and 600 K with a time step of 1 fs and a time step of 5 ps. It was observed that 3*R*-MoS_2_ also maintains the ABC stacking at temperatures between 300 K and 600 K. There is no significant displacement at the PA, and the changes in bond lengths and bond angles are not significant. The results demonstrate that intercalation in the PA is stable in AIMD simulations conducted at temperatures between 300 K and 600 K. Additionally, the Al^3+^ can diffuse rapidly into 3*R*-MoS_2_ due to the unaltered chemical bonding nature of the latter.

### 2.2. More Al^3+^ Inserted in 3R-MoS_2_

**Figure 2:**
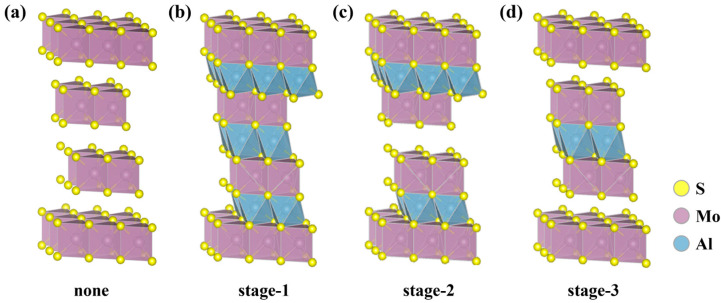
Schematic representations (side view) of the optimized structures of the four different intercalated stages with 0, 12, 8, 4 Al^3+^ inserted in 3*R*-MoS_2_: (**a**) none, (**b**) stage-1, (**c**) stage-2, and (**d**) stage-3.

As shown in Figure 2 and Figure S2, we simulated three different interpolation stages (stages 1–3), each using four different concentrations. 3, 6, 9 and 12 Al^3+^ are inserted in MoS_2_ for stage-1, 2, 4, 6 and 8 Al^3+^ are inserted in MoS_2_ for stage-2. 1, 2, 3 and 4 Al^3+^ are inserted in MoS_2_ for stage-3. A 3 × 3 × 2 supercell consisting of 24 S atoms and 12 Mo atoms was constructed for the calculations of stage-1, stage-2, and stage-3. It should be noted that the system was modeled using a pure 3*R*-MoS_2_ ABC-stacked structure comprising three MoS_2_ layers with an interlayer spacing of 2.80 Å. It is, therefore, believed that the MoS_2_ model is more suitable for simulating real experimental observations in ultra−fast AIBs.

It has been found that good cathode materials for AIBs require suitable interlayer spacing as well as reasonable Al binding strength. Therefore, MoS_2_ with these two characteristics happens to be a potential cathode for AIBs. We first investigated the structural deformation in MoS_2_ due to Al^3+^ inserted. There are three interlayer distances in the unit cell named Ind1, Ind2, and Ind3, shown in Figure S3. All distances are projected onto the C-axis and listed in Table 1. The layer spacing in pure MoS_2_ (2.80 Å), which is very close to the experiment data 2.98 Å and corresponds to the (006) diffraction plane of MoS_2_ (JCPDS No. 17–0744), is larger than the covalent radius of Al (1.18 Å) [22]. The results show that the interlayer spacing expands with the insertion of Al^3+^ at first for all the stages. All the expansion layers spacing are about 3.2 Å. This is due to the electrostatic interaction between Al and Mo ions. And the distance shrinks 0.1–0.5 Å for the layer without Al^3+^ inserted. After the interlayer spacing expands, further intercalation becomes smooth. Thus, with more Al^3+^ continuing to be embedded, the change in MoS_2_ layer spacing becomes smaller. It can also be considered that the insertion of Al^3+^ will not cause a large change in layer spacing, which verified that the structure of 3*R*-MoS_2_ is stable. The bonding of the Al and S atoms also prevents further expansion of the layer spacing. The increased interlayer spacing favors easier diffusion of Al^3+^ into MoS_2_. However, when 9 Al^3+^ was inserted in MoS_2_ in stage-1, and 6 Al^3+^ was inserted in MoS_2_ in stage-2, the lattice changed much (Figure S2), and the interlayer spacing varied irregularly. For example, when 9 Al^3+^ is inserted in MoS_2_ in stage-1, the Ind1 and Ind2 become smaller. This implies that the electrostatic interaction force is not uniform. However, the calculated expanded layer spacing for 3*R*-MoS_2_ (1.1 Å) is smaller than the one of 2*H*-MoS_2_ (1.9 Å), which indicates that the structure stability of 3*R*-MoS_2_ is better than the one of 2*H*-MoS_2_ [17,23].

### 2.3. Staging Mechanism

To further analyze the staging mechanism, the relative stabilities of stage-1, stage-2, and stage-3 with the same Al^3+^ concentration were compared. Divided into four sections, each inserted with the same concentration of Al^3+^, the optimized structures of stages 1, 2, and 3 are shown in Figure S4 and expressed in terms of relative energetics. The results show that stage-1 is more stable than stage-2 when the same concentration of 6 Al^3+^ is inserted, and stage-2 is again more stable than stage-3 when Al^3+^ is reduced to 4, and the same is true for subsequent reductions to 3 and 2. Thus, we believe that Al^3+^ prefers to insert into the galleries simultaneously rather than covering one gallery and then taking over the others. This is different from the mechanism of AlCl_4_^−^ insertion in graphene [24]. This phenomenon can be explained by the ion−ion Coulomb attraction. As Al ion and S ion bond to each other, it is believed that Al^3+^ first binds to S, and with the Al^3+^ increasing, the repulsion between Al and Mo atoms increases. Thus, Al^3+^ will not cover one layer and then take over the other. That will increase the repulsive force between Al and Mo atoms. As the concentration of Al^3+^ increases, the repulsive force also rises, which may lead to lattice distortion, such as the formation of 6 Al^3+^. Nevertheless, if Al^3+^ is distributed uniformly throughout all layers, it will result in a reduction in lattice distortion, such as the presence of 6 Al^3+^ in stage-1. It is, therefore, postulated that the intercalation mechanism follows stage-1.

### 2.4. Binding Energy

To further assess the stability of the intercalated compounds, the binding energies calculated for all stages are listed in Table 1, and Figure S5 shows the variation of binding energy as a function of the weight percentage (wt%) of Al^3+^ intercalation in MoS_2_ for all three stages. The calculated binding energies are observed to be negative in all cases, indicating that Al^3+^ is readily embedded in MoS_2_ within the context of this study. This phenomenon can be attributed to the smaller atomic size of Al^3+^ in comparison to the spacing of the MoS_2_ layers, which facilitates the insertion process. Moreover, the interaction between the intercalated Al^3+^ and the host MoS_2_ layer is sufficient to overcome the van der Waals forces between the MoS_2_ layers. In addition, the insertion of Al^3+^ in the already expanded MoS_2_ main channel is facilitated by higher concentrations. This is due to an increase in the average interlayer distance, which results in a reduction in van der Waals forces between the MoS_2_ layers. It can be demonstrated that the Al^3+^ binding energy in the bulk MoS_2_ decreases with the amount of Al^3+^ in the same stage. Furthermore, the lower stages (stage-1) are more stable than the higher stages (stage-2, stage-3) for a given concentration of Al^3+^.

### 2.5. Electronic Properties

**Figure 3:**
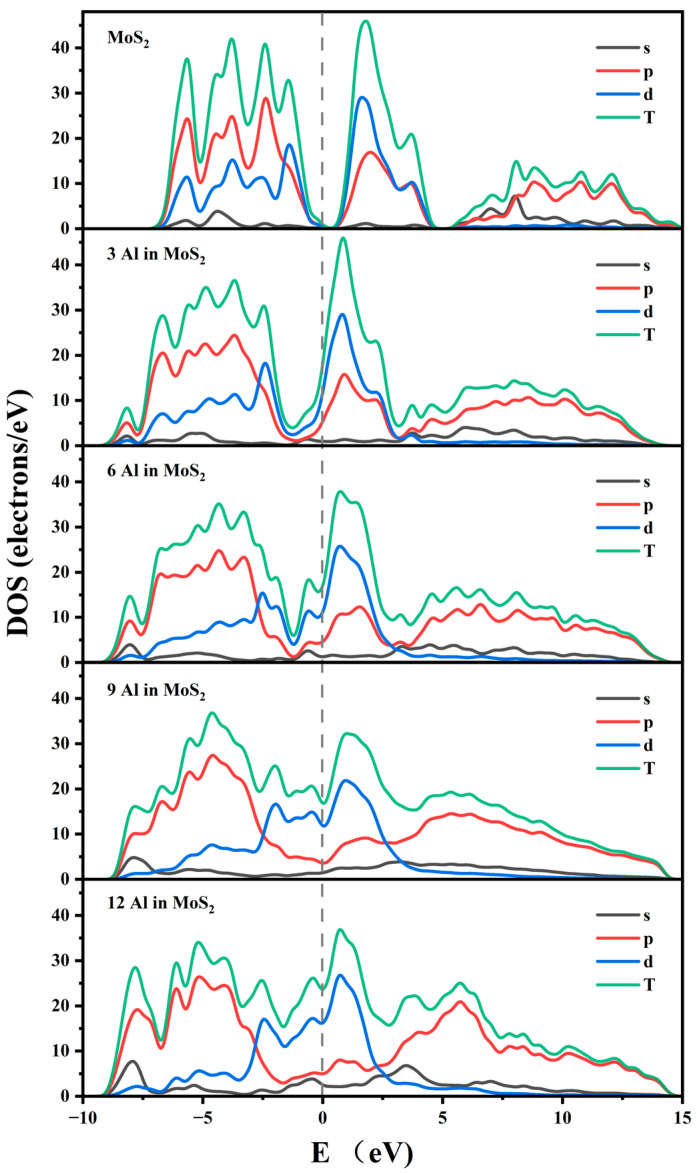
Total DOSs and partial DOSs of Al^3+^ intercalated 3*R*-MoS_2_ for stage-1. The Fermi level is set at zero, marked by the dashed line.

Conductive properties are very important for battery materials. The total density of states (TDOSs) and partial density of states (PDOSs) are calculated for Al^3+^ intercalated in MoS_2_ of stage-1, as shown in Figure 3. Only DOSs near the Fermi level are meaningful. Thus, the DOSs between −10 and 15 eV are shown in Figure 3. Furthermore, the DOSs at the Fermi level can effectively reflect the conductivity of the material [25]. It can be seen that with no Al^3+^ intercalating, 3*R*-MoS2 has poor electrical conductivity. Because there are fewer electrons at the Fermi level. And the electrons are mainly from Mo 4d and S 3p, which can be found in Figure S6a. The parts from −10 eV to 0 eV mainly are Mo 4d and S 3p hybridization, forming the S–Mo bond. With Al^3+^ intercalating, the DOSs at the Fermi level increase, which means the electronic conductivity increases. The electronic conductivity increases are mainly from Al 3p, Mo 4d, and S 3p orbitals. It is supposed that when Al^3+^ intercalates, the electrons transfer from Al 3p to S 3p. Correspondingly, the charge transfer of the Mo 4d is reduced, which can be verified from the Hirshfeld charge analysis. Thus, during charge/discharge, Al^3+^ increases the electronic conductivity of the materials.

Hirshfeld charge analysis is performed to understand the charge transfer more intuitively. From Figure S7, it was found that with the insertion of Al^3+^, the positive charge of Al increases and the charge of Mo decreases, whereas the Al metal is conductive and tends to lose its outermost electrons, which also leads to the transfer of electrons from the outermost layer of Al to S, resulting in the formation of Al–S bonds. And the electron transfer of Mo is weakening. Thus, the Mo–S bond is weakening. The electrons mainly transfer from Al 3p to S 3p orbitals. Furthermore, part electrons of Al 3s jump to Al 3p. The same goes for S atoms. Thus, both s and p orbitals of Al and S are hybridized and participate in bonding, which can be verified from Figure 3 and Figure S6b. The electrons transfer for Mo are mainly from Mo 5s to S 3p orbitals. And part electrons of Mo 5s jump to Mo 4d. Thus, there are fewer electrons left in the Mo 5s orbital. Only Mo 4d bond with S 3p, which agreed well with the analysis of the DOSs above. The sudden increase in Hirshfeld’s charge is due to lattice distortion for 9 Al^3+^ inserted in MoS_2_.

### 2.6. Diffusion of Al^3+^ in 3R-MoS_2_

**Figure 4:**
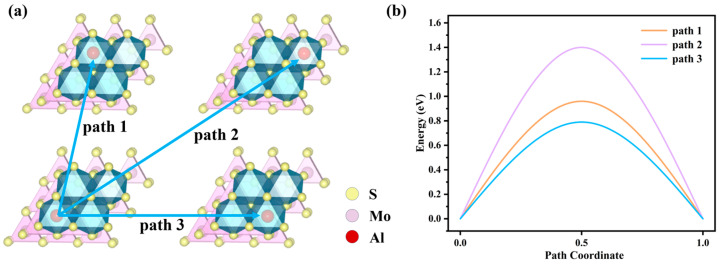
(**a**) Schematic representation of the diffusion barriers for the three pathways, (**b**) comparison of diffusion barriers for different paths.

The charging/discharging rate of batteries is related to the diffusion of the intercalated species through the electrodes. Therefore, to study the Al^3+^ diffusion mechanism in the 3*R*-MoS_2_ electrode, we have calculated barriers to finding energetically favorable diffusion paths. The PA site is the most stable. Hence, in this work, three favorable diffusion paths have been studied from a PA site to a neighboring A site. The first diffusion path, path 1, is from site PA to its nearest PA site, passing through a PB1 site (PA-PB1-PA); the second is path 2 involves the Al^3+^ diffusion from site A to another A site via site PB2 (PA-PB2-PA) and the third path, path 3, from site A to another nearest site A via site PB3 (PA-PB3-PA). All the diffusion paths and diffusion barriers are shown in Figure 4. From Figure 4, it is found that path 2 has the biggest barrier energy, as the diffusion path is the longest. Moreover, the barrier energies of path 1 and path 3 are closer and smaller than the ones of path 2. This can indicate that aluminum ions preferentially migrate to nearby stable positions. Moreover, the attraction of the S atom to Al^3+^ increased resistance and led to a higher barrier energy.

### 2.7. Electrochemical Properties of Al^3+^ Intercalated 3R-MoS_2_

The open circuit voltage (OCV) is a valuable measure of a battery’s performance. The open-circuit voltage (OCV) is evaluated by the equation below [26]:

(1) $$V=-(E_{x_{2}}-E_{x_{1}}-(x_{1}-x_{2})E_{Al})/3e(x_{1}-x_{2})$$

where $E_{x_{1}}$ and $E_{x_{2}}$ are the total energies of the Al_x_MoS_2_ at two adjacent stable systems of concentration *x*_1_ and *x*_2_, respectively.

**Figure 5:**
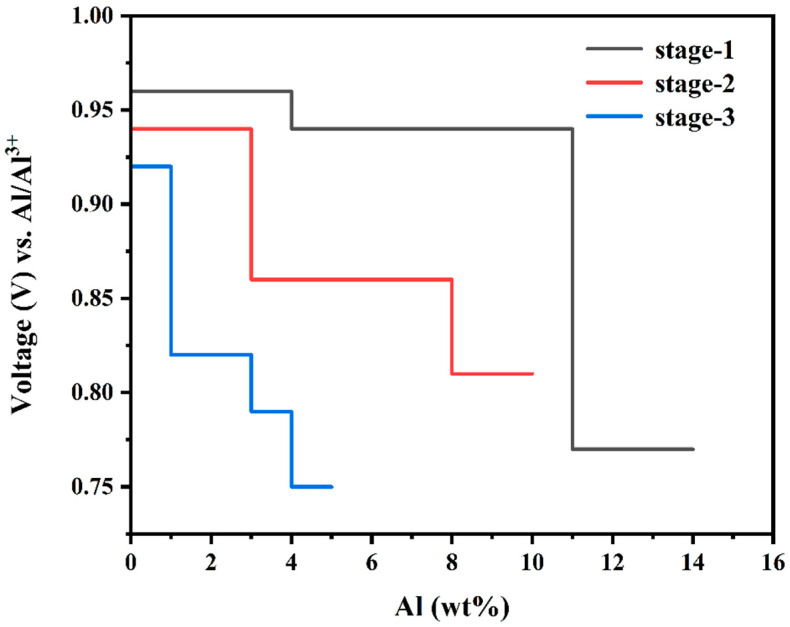
Voltage profile diagram of Al^3+^ intercalated 3*R*-MoS_2_ system against Al/A^3+^.

Figure 5 represents the variation of average voltage with the weight percentage (wt%) of Al^3+^ for all three stages (stages 1–3), and the calculated values for different stoichiometries are listed in Table 1. The OCV varies in a range of 0.77 V to 0.96 V for stage-1, 0.81 V to 0.94 V for stage-2, and 0.75 V to 0.92 V for stage-3. Since Al^3+^ are inserted into each layer at the same time in stage-1, the Coulomb and van der Waals forces of each layer need to be overcome, but stage-2 and stage-3 do not, which results in a higher voltage plateau in stage-1 relative to stage-2 and stage-3 [17,27,28].

The theoretical gravimetric capacity has been calculated using the following equation [29]:

(2) $$C=czF/M_{MoS_{2}}$$

where *c* is the number of inserted Al^3+^, *z* is the number of chemical valences of Al^3+^, *F* is the Faraday constant (26,801 mA h mol^−1^) and $M_{MoS_{2}}$ is the molar mass of the MoS_2_ cell. The calculated gravimetric capacities for stage-1, stage-2, and stage-3 are 502.30, 334.87, and 167.43 mAh g^−1^, listed in Table 2. From Table 2, it is obvious that the theoretical capacity of 3*R*-MoS_2_ stage-1 is higher than the experimental data of 2*H*-MoS_2_. Furthermore, the discharge plateau is observed to be higher than that observed in the other experiments. Therefore, based on our research and analysis, 3*R*-MoS_2_ is a potential cathode material for AIBs. The material’s stable layered structure enables it to maintain a long cycle life, high platform, and high capacity.

## 3. Computational Methods

The current calculations are based on Density Functional Theory (DFT) in the Cambridge Series of Total Energy Packets (CASTEP) plane wave code and on DS-PAW (Device Studio-Projected Augmented Wave) [33,34,35,36,37]. The Predew–Burke–Ernzerhof (PBE) generalized gradient approximation (GGA) was used in the description of the exchange–correlation potential. Norm-conserving pseudopotentials were used to describe the interaction of ionic core and valence electrons. Valence states were considered in this study corresponding to Al 3s^2^ 3p^1^ Mo 4d^5^ 5s^1^ and S 3s^2^ 3p^4^. After convergence tests (Figure S8), the cutoff energy for plane waves is eventually identified as 1820 eV. The d orbitals in the Mo ions have been localized using the Hubbard corrected GGA (GGA + U) method (U value of 4 eV). All structures are optimized by fully relaxing the atomic and lattice positions until the Hermann−Feynman force for all atoms is less than 0.01 eV Å^−1^. The numbers of K-points were set to 5 × 5 × 1. All systems are fully optimized, and the convergence criterion for the total energy is set to 5 × 10^−3^ eV. Van der Waals force interactions play an important role in the calculation of layered structures. Therefore, we have corrected the van der Waals force interactions using the DFT-3 method, which increases the potential energy and interatomic forces.

In this paper, a periodic structure was adopted. The unit cell of 3*R*-MoS_2_ is of the R3m space group and exhibits a hexagonal structure (Figure S3). The 2 × 2 × 2 supercell was employed in the calculations, which included 12 Mo atoms and 36 S atoms. Following the completion of geometry optimization, the resulting geometry constants are presented in Table S1. As evidenced by Table S2, the findings align with those of other experimental and theoretical studies [38,39,40].

To calculate the electronic structure, a 4 × 4 × 4 K-point sampling was employed within the Brillouin region. Ab initio molecular dynamics simulations (AIMD) were conducted using a regular ensemble (NVT) with a fixed number of ions, volume, and temperature. The AIMD simulations were configured to be conducted at temperatures spanning the range from 300 to 600 K, with a time step of 1 fs and a duration of 5 ps. The temperature was maintained using a Nose thermostat model. The transition state search utilizes the fully optimized initial and final structures with RMS convergence set to 0.01 eV Å^−1^. The activation barriers are calculated from the energy difference between the transition state and the initial state. The calculation of the diffusion potential incorporates corrections for entropy and zero−point energy. The binding energy after Al^3+^ intercalation into 3*R*-MoS_2_ was calculated using the following equation [24]:

(3) $$E_{binding}=\frac{E_{Al_{x}MoS_{2}}-E_{MoS_{2}}-xE_{Al}}{x}$$

where $E_{Al_{x}MoS_{2}}$, $E_{MoS_{2}}$, $E_{Al}$ are the total energy of MoS_2_ with *x* Al inserted, MoS_2_ and single Al^3+^.

## 4. Conclusions

This paper presents an investigation into the electrochemical properties of 3*R*-MoS_2_ and a discussion of its potential use as an AIB. The most stable configuration is one in which a single Al^3+^ is inserted into a PA octahedron comprising six S atoms. Moreover, the binding energy calculation revealed that the initial Al^3+^ insertion is random in the ABC stacking structure for 3*R*-MoS_2_. The AIMD simulation results indicate that 3*R*-MoS_2_ can retain a stable structure within a temperature range of 300 to 600 K. As the number of Al^3+^ inserted into 3*R*-MoS_2_ increases, it is observed that the ions are more likely to be inserted into interlayer sites simultaneously rather than occupying only one interlayer site. The insertion of Al^3+^ resulted in an increase in the conductivity of 3*R*-MoS_2_, accompanied by a transfer of electrons from Al atoms to S atoms, leading to the formation of Al–S ionic bonds. According to the calculation, the theoretical maximum specific capacity of Al^3+^ intercalated 3*R*-MoS_2_ reached 502.30 mAg h^−1^, and the average voltage of the intercalation was in the range of 0.75−0.96 V. Considering these findings, it can be concluded that 3*R*-MoS_2_ has great potential as a cathode material for AIBs.

## Figures and Tables

**Table 1:** Interlayer distance (Ind1, Ind2, Ind3), average open-circuit voltage (OCV), and binding energy (BE) per Al^3+^ for all stages with different content considered.

Stages	No. of Al	OCV (V)	BE (eV)	Ind1 (Å)	Ind2 (Å)	Ind3 (Å)
Stage-1	3	0.96	−2.87	3.16	3.19	3.16
	6	0.94	−2.85	3.13	3.19	3.31
	9	0.94	−2.83	2.42	2.51	3.26
	12	0.77	−2.71	3.23	2.86	3.15
Stage-2	2	0.94	−2.83	3.15	2.78	3.22
	4	0.86	−2.69	3.33	2.79	3.23
	6	0.86	−2.65	3.19	2.78	3.05
	8	0.81	−2.60	3.17	2.80	3.22
Stage-3	1	0.92	−2.76	2.78	3.21	2.78
	2	0.82	−2.61	2.79	3.15	2.75
	3	0.79	−2.53	2.77	3.21	2.78
	4	0.75	−2.46	2.81	3.18	2.77

**Table 2:** The calculated theoretical specific capacity compared with other data.

Cathode	Theoretical Capacity	Initial Capacity (mA h g^−1^)/ Current Density (mA g^−1^)	Cyclic Capacity (mA h g^−1^)/ Cycle Number	Discharge Plateau (V)	Ref.
stage-1	502.30	−	−	0.77–0.96	−
stage-2	334.87	−	−	0.81–0.94	−
stage-3	167.43	−	−	0.75–0.92	−
2*H*-MoS_2_	−	253.6/20	66.7/100	0.5–0.9	[17]
2*H*-MoS_2_/*N*-doped carbon	−	−	232/500	0.4	[30]
2*H*-MoS_2_-Maxene	−	224/-	166/60	0.3, 0.9	[28]
2*H*-MoS_2_/RGO	−	278.1/1000	161.1/100	−	[31]
2*H*-MoS_2_	−	249.6/1000	80.9/100	−	[31]
Spongy MoS_2_	−	214.2/500	129.5/1000	0.5	[23]
Bi_2_S_3_/MoS_2_	−	274.3/375	132.9/100	0.8	[32]

## Data Availability

Data are contained within the article and Supplementary Materials.

## Supplementary Materials

The following supporting information can be downloaded at: https://www.mdpi.com/article/10.3390/molecules29225433/s1, Figure S1: Molecular dynamics simulation analysis at different temperatures as a function of time step and the obtained structures, (a) 300 K, (b) 400 K, (c) 500 K and (d) 600 K; Figure S2: Schematic representations (side view) of the optimized structures of the three different intercalated stages with different Al^3+^ concentration: 12 (a) 9 (b) 6 (c) 3 (d) Al^3+^ inserted in 3*R*-MoS_2_ for stage-1, 8 (e) 6 (f) 4 (g) 2 (h) Al^3+^ inserted in 3*R*-MoS_2_ for stage-2, 4(i) 3(j) 2(k) 1(l) Al^3+^ inserted in 3*R*-MoS_2_ for stage-3; Figure S3: The 2 × 2 × 2 super-cell of 3*R*-MoS_2_ after geometry optimization; Figure S4: Systematic illustration of staging mechanism for (a) 6 Al^3+^ in stage-2 and stage-1, (b) 4 Al^3+^ in stage-3 and stage-2, (c) 3 Al^3+^ in stage-3 and stage-1, (d) 2 Al^3+^ in stage-3 and stage-2. RE (in eV) is the relative energetics for same concentrations; Figure S5: Binding energy per molecule for all stages of Al^3+^ as a function weight percentage of Al^3+^ in Al_x_MoS_2_; Figure S6: Total DOSs and partial DOSs of 3*R*-MoS_2_ (a) and 12 Al^3+^ intercalated 3*R*-MoS_2_ (b). The Fermi level is set at zero marked in dash line; Figure S7: Hirshfeld charge for stage-1; Figure S8: Convergence tests for the calculation of 3*R*-MoS_2_; Table S1: Lattice constants a, c and volume of 3*R*-MoS_2_ after geometry optimization compared with other experimental and theoretical data; Table S2: Lattice constants a, c, volume and binding energy of 3*R*-MoS_2_ with Al atom inserted in position A (top, middle and bottom position) and B (top, middle and bottom position).
