# Supplementary material for: First-Principles Study of 3R-MoS2 for High-Capacity and Stable Aluminum Ion Batteries Cathode Material
Source: Molecules. 2024 Nov 18;29(22):5433. doi: 10.3390/molecules29225433 (PMC11597766; doi:10.3390/molecules29225433)
Supplement: Supplementary file 1 [file molecules-29-05433-s001.zip › molecules-3279853-supplementary.pdf]

Supporting Information

First-Principles Study of 3R-MoS2 for High-Capacity and Stable Aluminum Ion Batteries Cathode Material

Bin Wang [1,2,3,*], Tao Deng [1,2], Quan Zhou [1], Chaoyang Zhang [1], Xingbao Lu [1] and Renqian Tao [4].

[1] School of Physics and Electronic Engineering, Xinxiang University, Xinxiang 453003, China.

[2] School of Mechanical Engineering, Chengdu University, Chengdu 610106, China.

[3] Henan Province Engineering Research Center of New Energy Storage System, Xinxiang University, Xinxiang 453003, China.

[4]. School of Physical Science and Technology, Lanzhou University, Lanzhou 730000, China

Corresponding author: Dr. Bin Wang, *wangbin2013@xxu.edu.cn*

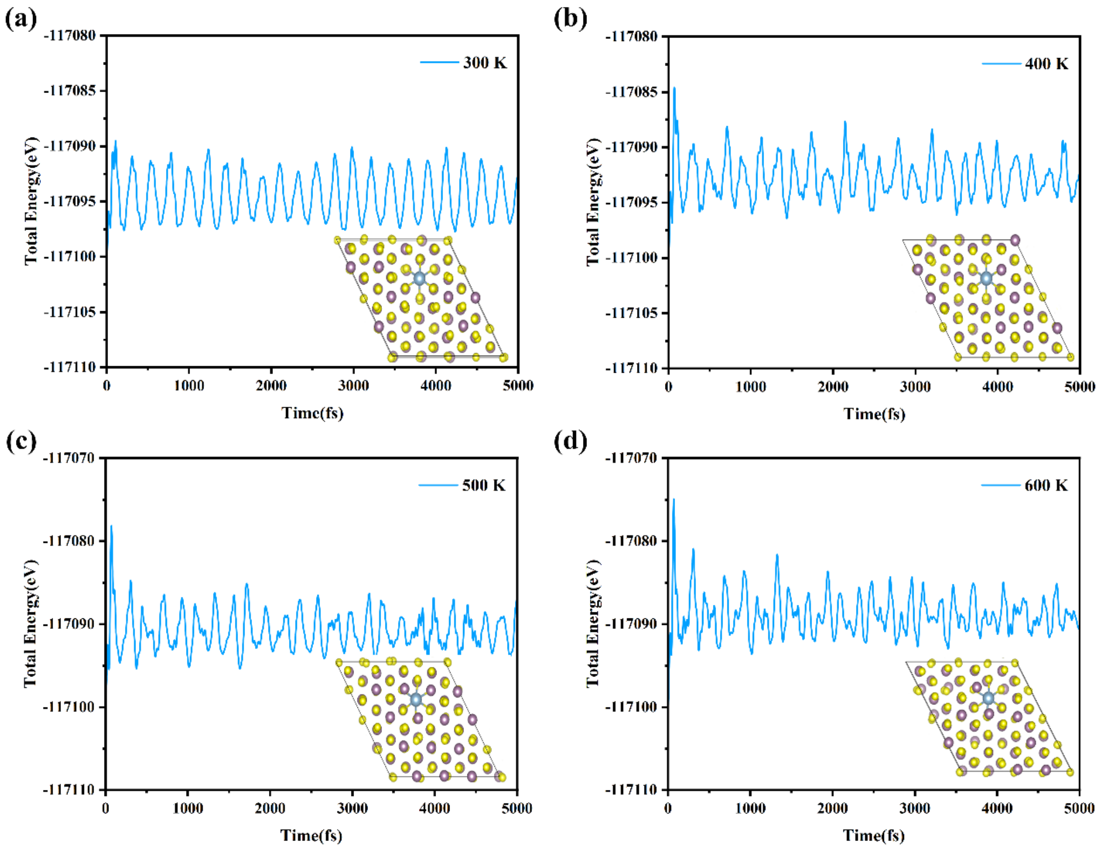

Figure S1. Molecular dynamics simulation analysis at different temperatures as a function of time step and the obtained structures, **(a)** 300K, **(b)** 400 K, **(c)** 500 K and **(d)** 600 K.

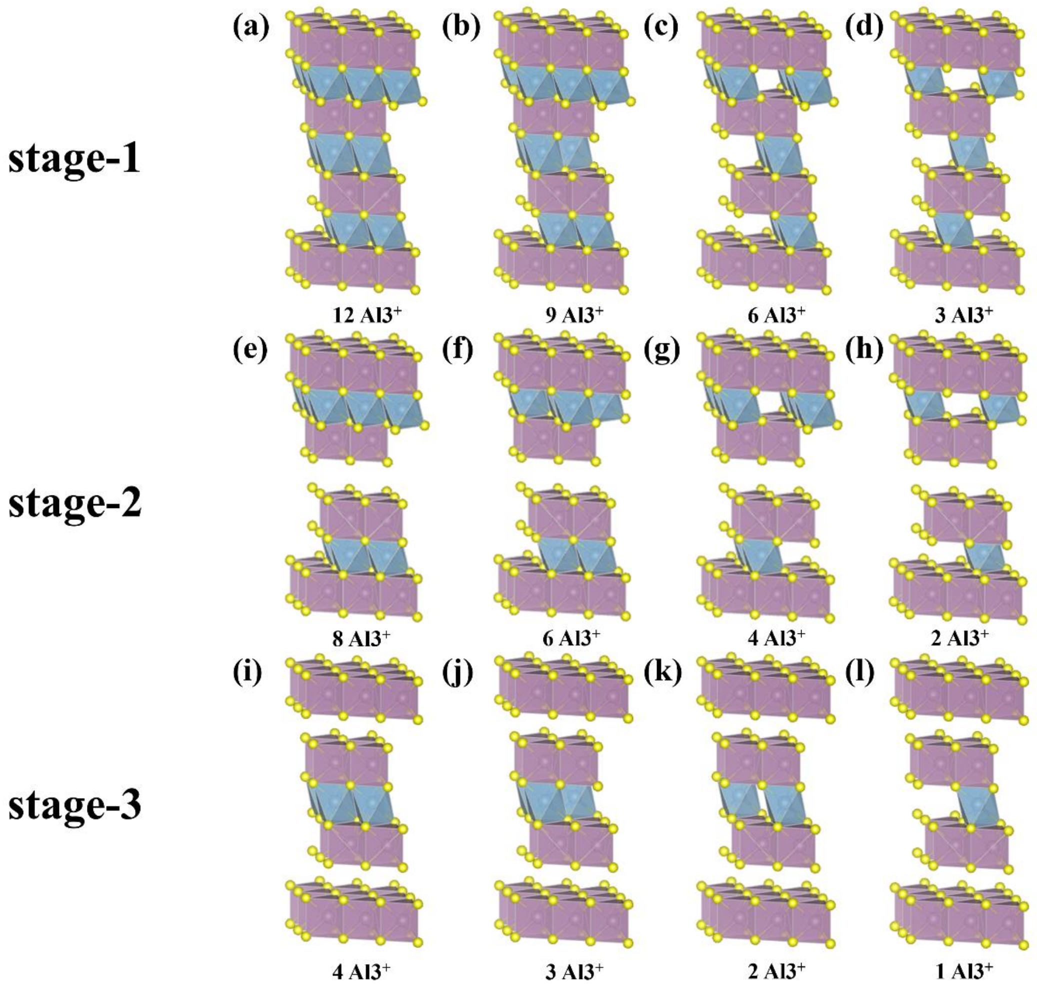

Figure S2. Schematic representations (side view) of the optimized structures of the three different intercalated stages with different Al^{3+} concentration: 12**(a)** 9**(b)** 6**(c)** 3**(d)** Al^{3+} inserted in 3*R*-MoS_2 for stage-1, 8**(e)** 6**(f)** 4**(g)** 2**(h)** Al^{3+} inserted in 3*R*-MoS_2 for stage-2, 4**(i)** 3**(j)** 2**(k)** 1**(l)** Al^{3+} inserted in 3*R*-MoS_2 for stage-3.

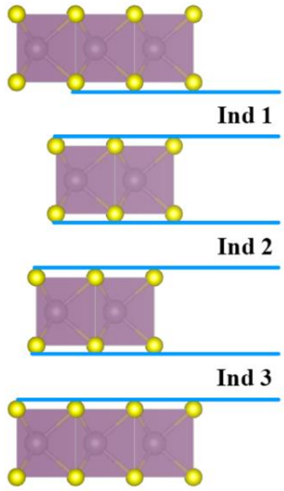

Figure S3. The 2×2×2 super-cell of 3R-MoS_2 after geometry optimization.

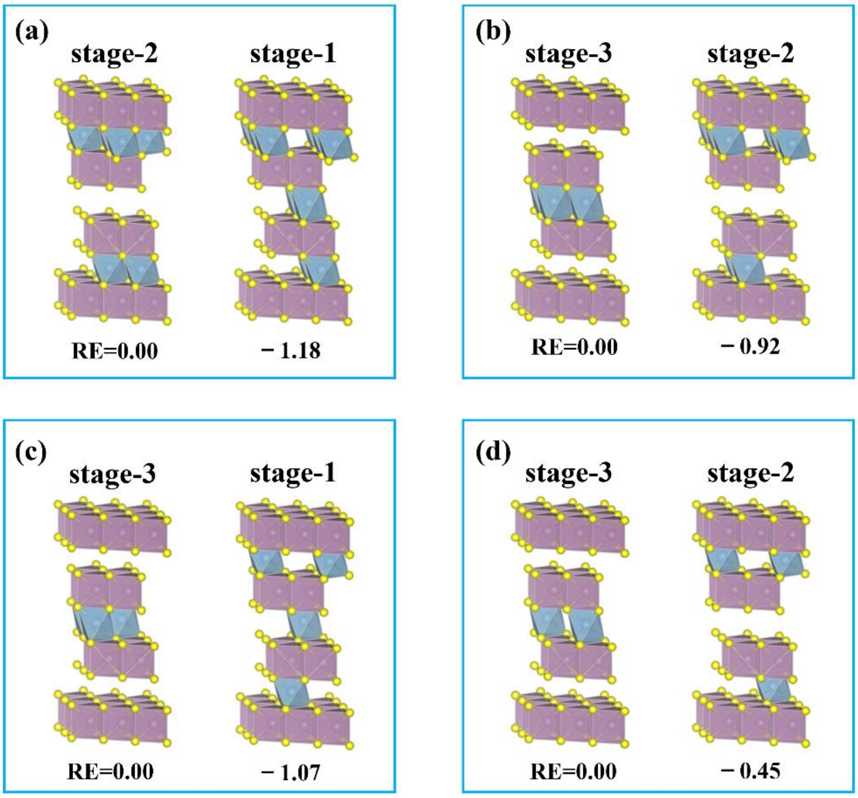

Figure S4. Systematic illustration of staging mechanism for **(a)** 6 Al^{3+} in stage-2 and stage-1, **(b)** 4 Al^{3+} in stage-3 and stage-2, **(c)** 3 Al^{3+} in stage-3 and stage-1, **(d)** 2 Al^{3+} in stage-3 and stage-2. RE (in eV) is the relative energetics for same concentrations.

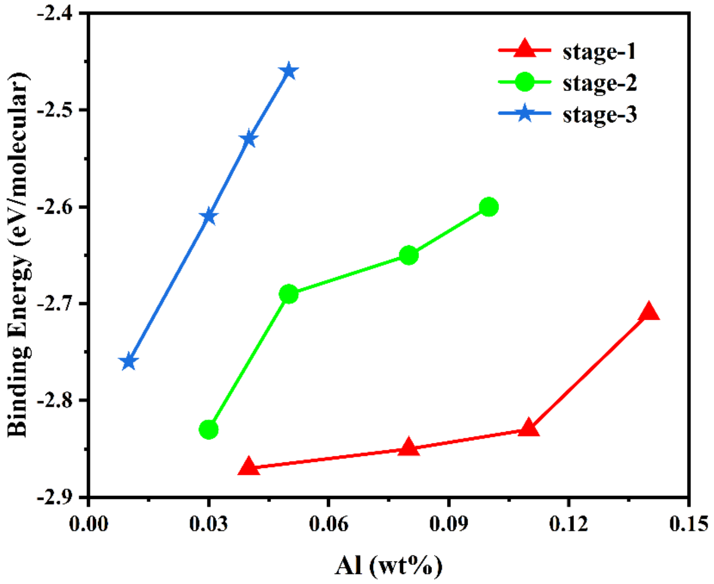

Figure S5. Binding energy per molecule for all stages of Al^{3+} as a function weight percentage of Al^{3+} in Al_xMoS_2.

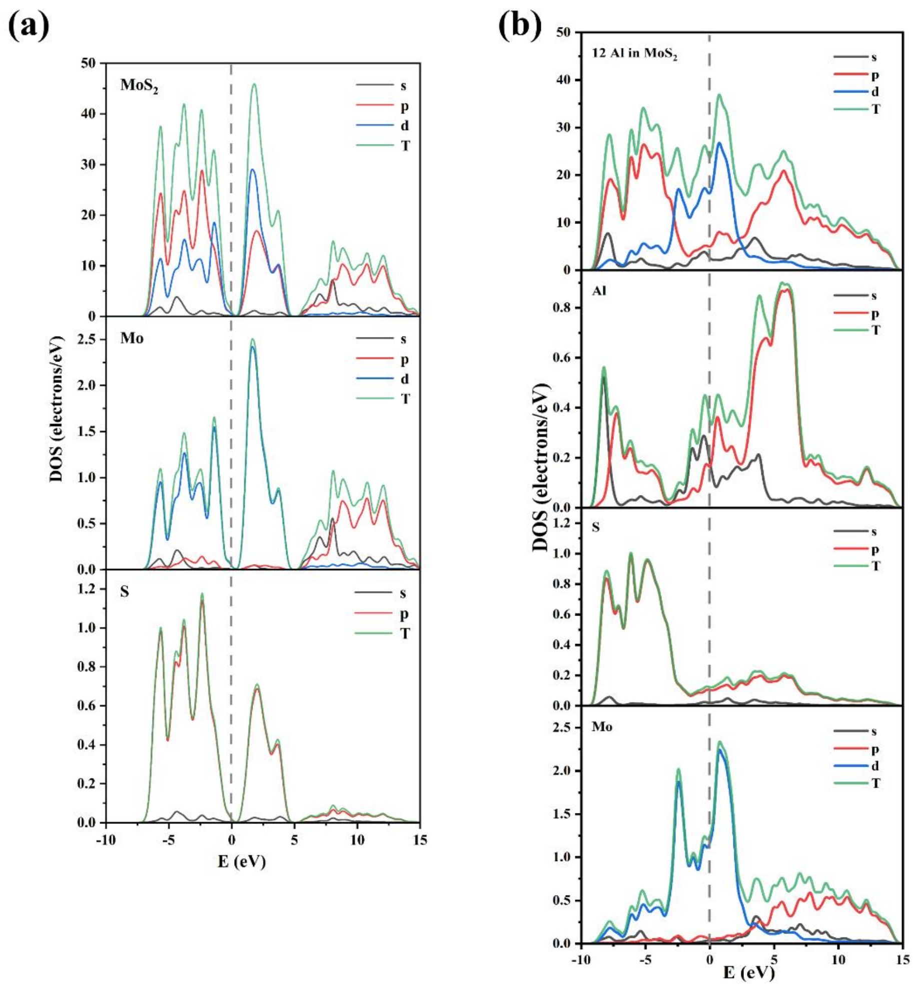

Figure S6. Total DOSs and partial DOSs of 3*R*-MoS_2 **(a)** and 12 Al^{3+} intercalated 3*R*-MoS_2 **(b)**. The Fermi level is set at zero marked in dash line.

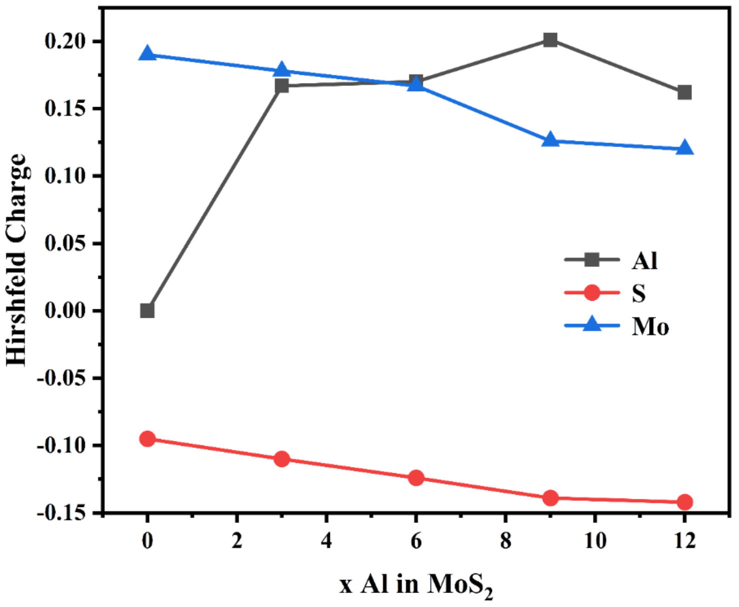

Figure S7. Hirshfeld charge for stage-1.

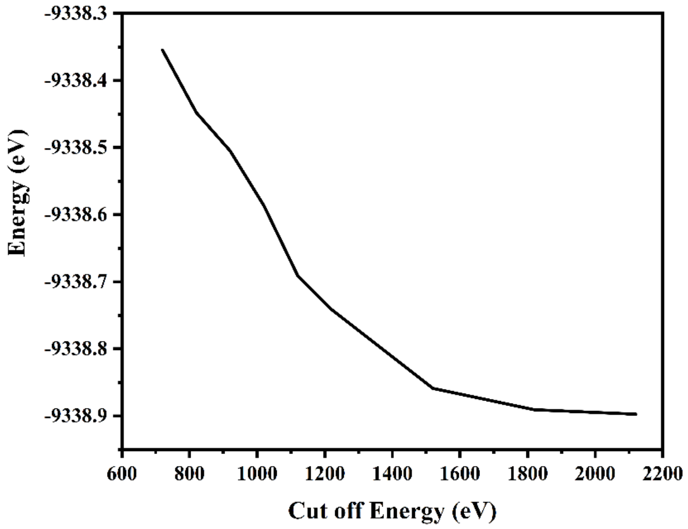

Figure S8. Convergence tests for the calculation of 3*R*-MoS_2.

Table S1. Lattice constants a, c and volume of 3*R*-MoS_2 after geometry optimization compared with other experimental and theoretical data.

	a(Å)	c(Å)	V(Å^3)
present	3.185	17.85	157.09
Exp.**[1]**	3.17	18.38	159.95
Exp. **[2]**	3.16	18.37	159.16
Theo. **[3]**	3.20	20.14	178.57

Table S2. Lattice constants a, c, volume and binding energy of 3*R*-MoS_2 with Al atom inserted in position A (top, middle and bottom position) and B (top, middle and bottom position).

	a(Å)	c(Å)	V(Å^3)	$E_{binding}$
PA top	6.38	18.32	645.72	-2.22
PA middle	6.38	18.32	645.75	-2.22
PA bottom	6.38	18.32	645.71	-2.22
PB top	6.39	18.69	660.30	-1.63
PB middle	6.39	18.69	660.30	-1.63
PB bottom	6.39	18.68	660.15	-1.62

Reference

1. Jellinek, F.; Brauer, G.; Müller, H. Molybdenum and Niobium Sulphides. *Nature* **1960**, *185*, 376–377
2. Schönfeld, B.; Huang, J.J.; Moss, S.C. Anisotropic Mean-Square Displacements (MSD) in Single-Crystals of 2H- and 3R-MoS2. *Acta Cryst. B* **1983**, *39*, 404–407.
3. Coutinho, S.S.; Tavares, M.S.; Barboza, C.A.; Frazão, N.F.; Moreira, E.; Azevedo, D.L. 3R and 2H Polytypes of MoS2: DFT and DFPT Calculations of Structural, Optoelectronic, Vibrational and Thermodynamic Properties. *J. Phys. Chem. Solids* **2017**, *111*, 25–33.
